# Editorial: RNA machines

**DOI:** 10.3389/fgene.2023.1290420

**Published:** 2023-09-27

**Authors:** Nikolay E. Shirokikh, Kirk Blomquist Jensen, Nehal Thakor

**Affiliations:** ^1^ The John Curtin School of Medical Research, The Australian National University, Canberra, ACT, Australia; ^2^ School of Biological Sciences, Faculty of Sciences, University of Adelaide, Adelaide, SA, Australia; ^3^ Department of Chemistry and Biochemistry, University of Lethbridge, Lethbridge, AB, Canada

**Keywords:** RNA, splicing, non-coding RNA, short non-coding RNA, RNA translational control, RNA viruses, RNA synthetic biology, ribozymes

## The omnipotent RNA machines

RNA has been escaping the limelight for decades while it was used to decipher the genetic code (Gardner et al., 1962; Nirenberg et al., 1965; Nirenberg, 2004), establish the rules of complementary nucleotide pairing (Magasanik et al., 1950), develop sequencing techniques (Jou et al., 1972), showcase foundational biomolecule interactions (Fox et al., 2018; Tauber et al., 2020) and assist in protein production (Spirin et al., 1988). The central dogma of molecular biology, an information flow typical to all living things, has been conceived to be centred around RNA (Boivin and Vendrely, 1947). Yet even now, we often employ a term “encoded in the DNA”, whereas the actual code is written in the RNA and its decoding is also performed by the ancient RNA molecular machinery of the cells. Notably, newer molecule types, classes and functions consistently emerge and re-emerge in the RNA world. The expanding diversity of RNA modifications and the new varieties of “nonocoding” but information-rich RNA remain as a vast and constantly replenisheable reservoir of biologically active molecules (Mercer et al., 2009; Roundtree et al., 2017). RNA now has been intertwined in more and more intricate cellular and viral processes and activities, tying it into the majority–perhaps, all–of the biological pathways.

## Refinement of splicing by and for RNA

A fundamentally distinctive feature of the RNA is its versatility. Consequently, we find the involvement of RNA function across all stages of gene expression. In eukaryotic and some prokaryotic cells, RNA-determined splicing is one of the cornerstone processes of gene expression. Splicing is driven by introns which likely originate from ancient retroelements (RNA retroviruses derivatives) but now are tightly controlled in the cells (Xiong and Eickbush, 1990; Flavell, 1995; Boeke and Stoye, 1997; Koonin, 2006; Hoskins and Moore, 2012; Zimmerly and Semper, 2015). Regulation of intron activity during splicing, such as in alternative splicing, creates an extra layer of diversity which is evolutionarily malleable and is thought to be extremely important in cell differentiation of complex organisms such as vertebrates, and in complex organs such as the brain (Santoni et al., 1989; Hoskins and Moore, 2012; Braunschweig et al., 2013; Irimia et al., 2014; Weatheritt et al., 2016; Ha et al., 2021). Interrupted genes themselves and splicing have been discovered relatively early in eukaryotic viruses, and then in the genomes of eukaryotic and some prokaryotic cells (Berget et al., 1977; Chow et al., 1977; Darnell, 1978; Keller and Noon, 1984; Apirion and Miczak, 1993; Belfort et al., 1995; Herbert and Rich, 1999; Benler and Koonin, 2022; Vosseberg et al., 2022). The constituents and structures of the major (type I) eukaryotic splicing machinery are well-established, and include the small nuclear ribonucleoproteins (snRNPs) U1, U2, U4, U5 and U6, and a number of auxiliary protein factors (Wahl et al., 2009; Will and Lührmann, 2011; Zhao and Pyle, 2017; Kastner et al., 2019; van der Feltz and Hoskins, 2019). The action of snRNA-organised spliceosomal complexes on the introns of nuclear transcripts involves first complex E, where U1 binds to the GU sequence at the 5′splice site donor, U2 Auxiliary Factor 1 binds to the AG sequence at the 3′splice site acceptor, U2 Auxiliary Factor 2 binds polypyrimidine tract between the branch point and 3′splice site, and Splicing Factor 1 binds to the branch point. A series of rearrangements then occurs towards Complex A with U2 binding the branch point, Complex B with U4, U5 and U6 joining and releasing U1, and Complex C with U4 release, lariat formation catalysis by U2 and U6 and then exon ligation (Rogers and Wall, 1980; Maniatis, 1991; Stamm et al., 2005; Wahl et al., 2009; Wang et al., 2015; Kastner et al., 2019). Manuel et al. summarise the impact of alternative splicing on the proteome diversity in a comprehensive review also describing splicing contribution to the generation of noncoding and circular RNAs. They address the important challenges in the prevalence of functional alternative splicing, the artefact-prone difficulties it creates in genome annotations, and the linking between observed RNA splice-types and peptide datasets, including advances and limitations of the current technologies.

Splicing picture was not complete until the alternative “minor” (type II) splicing apparatus has been identified (Tarn and Steitz, 1996; Sharp and Burge, 1997). Initially considered as indeed a “minor” variation that rarely occurs with efficiency and operates with a near-complete own set of snRNPs (U11, U12, U4atac, and U6atac, while U5 remains the same), AU-AC or GU-AG acceptor and donor definitions and the branch sequence, U12-type splicing has been found much more prolific (Park et al., 2016; Moyer et al., 2020; Fast, 2021). While not universally encountered, type II spliceosone and its introns are remarkably conserved (human genome harbours about 700 U12-type introns, but it can be much more (Larue et al., 2021)) and were recently highlighted interacting with the type I splicing machinery in diverse ways. In an insightful review, Akinyi and Frilander enlist cooperation between the U2 and U12 types using the example of *SNRNP48* and *RNPC3* genes (encoding U11 and U12 proteins) where feedback-stabilised U11 and U12 interactions lead to the recognition of 3′splice site 1 and synthesis of a non-productive, nuclear-retained transcript–as opposed to the use of 3′splice site 2 that results in cytoplasmic export and translation. They further showcase examples of direct competition between U2 and U12 types (for *HNRNPLL*, *ZNF207*, *C1orf112*, *NCBP2*, *PRMT1*, *dZRSR2/Urp*, *CTNNBL1*, *CUL4A*, *SPAG16*, *Prospero*, *SRSF10*, *MAPK8/9*, *TMEM87a/b*, *CENATAC* and other genes), or cryptic activation of U2 splicing in a deficit of U12 machinery (for *SNRPE*, *RCD8/EDC4*, *SLC9A8*, *MAPK12*, *LKB1* and other genes), and discuss implications in the context of spliceosomal diseases including Peutz-Jager Syndrome, spondyloepiphyseal dysplasia tarda and Cerebral palsy.

Remarkably, splicing has always been linked to RNA polymerase II transcriptional dynamics (Barrass et al., 2015; Naftelberg et al., 2015; Herzel et al., 2017; Milligan et al., 2017; Ragan et al., 2019). In a similarity to the multitude of transcriptional regulators, splicing is modulated by an array of non-constitutive protein factors (Lin and Fu, 2007; Sapra et al., 2009; Änkö et al., 2012; Vuong et al., 2016). Previously, many of such regulators have been implicated in the production of unusual RNA types such as micro and circular RNA (Melamed et al., 2013; Conn et al., 2015; Salzman, 2016; Eger et al., 2018; Pillman et al., 2018; Ratnadiwakara et al., 2018), cases of alternative processing and splicing of micro-exons (Ustianenko et al., 2017; Torres-Méndez et al., 2019; Head et al., 2021), and are known contributors to the physiologically significant development, differentiation and pathogenesis processes (Irimia et al., 2014; Mochizuki et al., 2021). In an original phylogenetic research article, Huang et al. feature the conservation and diversity of SYF2, an important splicing factor that interacts with cyclin D-type binding-protein 1, a cell cycle regulator at the G1/S transition. SYF2 has been characterised as essential or stimulatory in a variety of proliferative situations, including cancer (Guo et al., 2014; Yan et al., 2015; Zhang et al., 2015). Huang et al. *d*emonstrate conservation of the phylogenetic and splicing patterns of SYF2 in animals, while its mRNA abundance patterns were substantially different across the different tissues of mammals. They demonstrate SYF2 is associated with the occurrence of cancer in breast, lung, spleen and reproductive organs, making SYF2 and its RNA interactors valuable therapeutic targets.

## RNA regulators conducting without a code

To act, RNA not necessarily needs to be decoded. Micro RNA and various noncoding RNA functions have been prominent in the transition space from transcriptional to post-transcriptional control and translation (Mehler and Mattick, 2007; Mercer et al., 2009; Änkö and Neugebauer, 2010; Schonrock et al., 2012; Salmanidis et al., 2014; Statello et al., 2021). With the advent of various high-throughput sequencing technologies, we began to substantially broaden the horizons of our understanding towards the “rare” RNA transcript type diversity (Kapranov et al., 2007; Ma et al., 2013; Gil and Ulitsky, 2020). Noncoding RNA of various types have emerged as functionally active in determining cell differentiation and development (Fatica and Bozzoni, 2014). Ni et al. dive into the classification of Terminus-Associated Non-coding RNAs (TANRs; as well as mRNA 5′-end associated noncoding RNAs) in an insightful review of these emerging RNA types. They highlight Terminus-Associated Short Non-coding RNAs (TASRs) and their antisense (aTASRs) varieties, Transcription Termination Site Associated RNAs (TTSa-RNAs), Transcription Boundary-Associated RNAs (TBARs), Terminus-Associated Long RNAs (TALRs) and 3′UTR-associated RNAs (uaRNAs), and explore evidence for their biogenesis, including the same and independent promoter models. Ni et al. further enlist organisation and discovery technology of several prominent TANRs and summarise the demonstrated TANR functions, including transcriptional interference, promoter and terminator juxtapositioning, transcription termination assistance, micro RNA sponging and sequence-directed RNA cleavage and modification. They note that functionally-relevant TANRs can originate also from long noncoding RNA genes, with *MALAT1* prominently exemplified by its translation-activating MALAT1-associated small cytoplasmic RNA (*MascRNA*) (Wilusz et al., 2012).

While individual micro RNAs may appear as less multifaceted regulators and interactors compared to the longer non-coding RNAs, a developing view is that micro RNAs function in networks, collectively targeting the entire pathways of the cells (Gao, 2008; Ryan et al., 2010; Bracken et al., 2016; Dragomir et al., 2018). Such networks offer a high degree of versatility, sophistication and accuracy of control. An interesting expansion of this idea is presented in a review of Budrass et al. where they thoroughly describe a new micro RNA regulation network and intersect it with the protein control network of a matching complexity, as found for the chaperones Heat Shock Protein 40s (Hsp40s; often referred to as J-proteins by their encoding *DNAJ* genes) (Laufen et al., 1999; Han and Christen, 2003). J-proteins are extremely conserved (from bacteria to human) and function as a “tailoring kit” for situationally activating Hsp70 proteins that have far less client discrimination. J-proteins are devise (over 40 in humans) and possess specific client binding, localisation and additional enzymatic activities (Cyr et al., 1994; Jiang et al., 2019). Budrass et al. review micro RNA target site predictions in J-protein mRNAs, demonstrate their conservation across mammals as well as vertebrates, and intriguingly showcase co-targeting of certain J-protein mRNAs (including *DNAJ A1, A2, B1, B4, B5, B6b, B9, C13, C21,* and *C23*) by micro RNAs of identical and different families, at one or multiple sites, opening new area of complex combinatorial regulatory opportunities.

## Translation of RNA and damage control

Decoding of the messenger(m)RNA into the proteins is the most crucial function performed by the RNA (Topisirovic and Sonenberg, 2011; Hershey et al., 2012; Shirokikh, 2022). Translational control is involved in nutrient, stress condition and damage sensing (Holcik and Sonenberg, 2005; Bramham et al., 2016; Ross et al., 2018; 2019; Janapala et al., 2019; Xie et al., 2019). Translation employs the most complete repertoire of RNA activities, including direct basepairing interactions, complex structure formation, functionally modified nucleotides, energised intermediates such as the aminoacyl-tRNA, precise macromolecular interactions as happens in the ribosomal subunit binding and elongation cycle dynamics, and ribozyme catalysis in the ribosomal peptidyl transferase centre. Translation initiation is the most responsive protein biosynthesis regulator in eukaryotes (Kozak, 1992; Pisarev et al., 2005; Sonenberg and Hinnebusch, 2009; Archer et al., 2016; Shirokikh and Preiss, 2018), and within it the accuracy of start codon recognition is especially important, whereby a small mistake can lead to misfolded proteins and adverse cell effects including malignancy (Fekete et al., 2005; Lomakin et al., 2006; Cheung et al., 2007; Asano, 2014; Thakur et al., 2020; Gleason et al., 2022). Accurate start codon recognition involves protein initiator tRNA “carriers”—of which a GTPase eukaryotic translation initiation factor 5B (eIF5B) is the most conserved, having its bacterial and archaeal counterparts (Ross et al., 2018; Shirokikh and Preiss, 2018). In an immersive mini-review, Chukka et al. discuss eIF5B at the crossroads the ribosomal, transfer and messenger RNA interactions. They highlight canonical and most conserved eIF5B functions in initiator tRNA stabilisation on the ribosomal small subunit and subunit joining. Chukka et al. also provide an outlook into the less obvious eIF5B activities in checkpointing eukaryotic small subunit maturation, conveying initiation with the alternative initiator tRNA carrier eIF2A active in certain stresses and interacting with viral (*e.g.*, hepatitis C virus and classical swine fever virus) and cellular (*e.g.*, X-linked inhibitor of apoptosis) internal ribosome entry sites (IRESes). eIF5B action in upstream Open Reading Frame (uORF)-regulated genes is reviewed and its overall cell survival-promoting and thus, malignancy-maintaining role is brought into the focus as an attractive drug target.

Another interesting activity of RNA tightly linked to translation is that of a cellular protection and damage sensing. Oxygenation and oxidative environments present a substantial challenge to the nucleic acids-based life, and especially so to the RNA which can become oxidised in a diverse ways. It has been known that oxidised RNA induces translational errors and may be neurodegenerative or carcinogenic (Tanaka et al., 2007; Fimognari, 2015; Guo et al., 2020). Seixas et al. thoroughly present types of oxidative RNA damage in a mini review covering the most ubiquitous oxidating agents, RNA injuries including 8-oxo-7,8-dihydroguanosine, 8-oxo-7,8-dihydroadenosine, 5-hydroxycytosine and 5-hydroxyuridine, and their effects on mRNA, tRNA, ribosomal and micro RNA function. Seixas et al. emphasise the known passive (scavenging) and active (repair) RNA injury protection systems in prokaryotes noting open questions in comprehensive identification of these components across all life.

## Multifunctional RNA in viruses and synthetic biology

RNA is often multifunctional in viruses where there can be certain restrictions on genome size, and in synthetic biology designs where vector and delivery limitations apply to the RNA length, together with the considerations of economy and cost (Afonin et al., 2014; Rossetto and Pari, 2014; Dao et al., 2015; Richert-Pöggeler et al., 2021). From its discovery, Human Immunodeficiency Virus 1 (HIV-1) has been intriguing the researchers with the multitude of functions of its highly-structured RNA modules, containing proteins in all three open reading frames and often with an overlap (splicing-, scanning-, frameshifting- and shunting-controlled), which can be synthesised from the 5′cap or IRES (Ohlmann et al., 2014; Guerrero et al., 2015; Reitz and Gallo, 2015; De Breyne and Ohlmann, 2019). In the DNA-integrated form the HIV-1 provirus can produce partially-spliced and fully-spliced transcripts, among the latter the *tat* mRNA, encoding the essential Tat transcriptional regulator of the virus. All viral transcripts share the initial 289 nt and thus the 59 nt of the highly structured *trans*-activation response (TAR) RNA element. TAR controls host translation via activation and suppression of Protein Kinase RNA-activated (PKR), and further activates viral transcription upon Tat binding (Guerrero et al., 2015). Inspired by their recent discovery that *tat* can have an IRES element active in latent infection (Khoury et al., 2020), Khoury et al. in an original research article embarked on a *tour de force* to explore cellular proteins possibly interacting with *tat* and modulating it*.* Discovering 243 significantly interacting proteins by *tat*-3×MS2-stem-loop-directed pull-down and mass spectrometry in latent and productive T-cells, they used knockdown of several top hits to identify Signal Recognition Particle 14 (SRP14) and High-mobility group box 3 (HMGB3) proteins affecting HIV infection the most. Using RNA modification protection, Khoury et al. located the SRP14 and HMGB3 binding sites nearby the *tat* start codon. Most intriguingly, SRP14 and HMGB3 negatively regulated latent and productive infection, while stimulating and repressing Tat synthesis, respectively. Khoury et al. propose SRP14 and HMGB3 alter the efficiency of the *tat* IRES, opening new depths in the lentiviral host interactions and additional pathways to manipulate HIV reactivation.

Interestingly, HIV-1 RNA may contain other RNA regulators, riboswitches (Ooms et al., 2004; Boeras et al., 2017). Riboswitches are compact structural modules of RNA conditionally obstructing (or promoting) a certain process (Garst et al., 2011; Breaker, 2012). Riboswitches can be sensitive to an interaction with another macromolecule or a small compound, or physical conditions such as temperature, pH, salinity, *etc.,* (Mironov et al., 2002; Nahvi et al., 2002; Serganov and Nudler, 2013). Riboswitches can be placed ahead of an “amplifying” stage of a synthetic construct expression, such as transcription or translation, and thus are among the most interesting synthetic biology tools (Breaker, 2018; Kavita and Breaker, 2022). In a brief research report, Korniakova et al. present a new fluoride-sensitive vector design incorporating a fluoride riboswitch (Ren et al., 2012) in the reporter 5′UTR, downstream of the testable promoter region. The plasmid allows to decouple cloning of powerful and potentially cell-damaging promoters from their functional testing, while maintaining same arrangement of the vector for the ease of cloning and comparisons.

## Perspectives for RNA as a molecular machine of design

It may not be an overstatement to name the RNA an ultimate molecular machine of life. RNA often performs in relatively straightforward ways built on direct molecular recognition through tertiary structure and basepairing, as happens during micro RNA target binding, and in distinct enzymatic reactions, as occurs during the intron lariat formation and excision. In the other cases, RNA performs as the structural and enzymatic core of conveying molecular machines such as the ribosome, where it uses chemical energy to process, transform and realise biological information. It is quite remarkable that the RNA can “work” with all types of biological macromolecules, be an enzyme and a substrate, carry and decode the genetic information, signal, receive and operate with chemical potentials, making it a “complete”, self-sufficient molecule. This self-sufficiency contains a value for novel synthetic biology designs, that is, being recognised in RNA vaccines and gene replacement therapeutics of more sophisticated construction, such as self-amplifying RNA (Rodríguez-Gascón et al., 2014; Brito et al., 2015; Pardi et al., 2018; Dolgin, 2021). It also contains a substantial combinatorial challenge of finding an optimal function in a sea of interactions and activities. We can hope to keep learning from extant (and extinct) life to identify new elements of RNA control, and employ approaches based on artificial intelligence to devise new RNA modules and their applications (Lv et al., 2021; Mohanty and Mohanty, 2021). Thankfully and as exemplified in this Research Topic, we cannot stop to continuously discover new, sometimes unexpected, nuances of the RNA-based processes.
